# Advanced Thermal Protection Systems Enabled by Additive Manufacturing of Hybrid Thermoplastic Composites

**DOI:** 10.3390/polym17222974

**Published:** 2025-11-07

**Authors:** Teodor Adrian Badea, Alexa-Andreea Crisan, Lucia Raluca Maier

**Affiliations:** Composite Materials Laboratory for Aeronautical Field, Romanian Research & Development Institute for Gas Turbines-COMOTI, 220D Iuliu Maniu Av., 061126 Bucharest, Romania; teodor.badea@comoti.ro (T.A.B.); raluca.maier@comoti.ro (L.R.M.)

**Keywords:** ablative protection system, additive manufacturing, fiber-reinforced polymers, hybridization, oxy-acetylene torch testing

## Abstract

This study investigates seven advanced hybrid composite thermal protection system (TPS) prototypes, featuring an innovative internal air chamber design that reduces heat conduction and enhances overall thermal protection performance. Specimens were manufactured by fused deposition modeling (FDM), an additive manufacturing technique, using a fire-retardant thermoplastic. Selected configurations were reinforced with continuous carbon or glass fibers, coated with ceramic surface layer, or hybridized with carbon fiber reinforced polymer (CFRP) layers or a CFRP laminate disk. To validate performance, a harsh oxy-acetylene torch (OAT) protocol was implemented, deliberately designed to exceed the severity of most reported typical ablative assessments. The exposed surface of each specimen was subjected to direct flame at a 50 mm distance, recording peak temperatures of 1600 ± 50 °C. Two samples of each configuration were tested under 60 and 90 s exposures. Back-face thermal readings at potential payload sites consistently remained below 85 °C, well under the 200 °C maximum standard threshold for TPS applications. Several configurations preserved structural integrity despite the extreme environment. Prototypes 4.1 and 4.2 demonstrate the most favorable performance, maintaining structural integrity and low back-face temperatures despite substantial thickness loss. By contrast, specimen 6.2 exhibited rapid degradation following 60 s of exposure, which served as a rigorous and selective early-stage screening tool for evaluating polymer-based ablative TPS architectures.

## 1. Introduction

The rapid advancement of hypersonic vehicles has intensified focus on thermal protection, where heat management remains a critical bottleneck [1]. Polymer nanocomposites with inorganic nanomaterial additives exhibit superior thermal resistance, flame resistance, chemical resistance, conductivity, and reduced permeability. Ongoing research emphasizes applications in rocket nozzle ablatives, thermo-oxidatively resistant carbon–carbon composites, and damage-tolerant epoxy matrices [2]. Polymeric ablatives (PAs) are the most versatile thermal protection system (TPS) materials, with advances such as the Phenolic Impregnated Carbon Ablator (PICA) developed by the National Aeronautics and Space Administration (NASA) and the modified version PICA-X developed by Space Exploration Technologies Corporation (SpaceX), with the latter offering improved ablation resistance at a one-order-of-magnitude lower cost [3]. Ablative TPS materials, particularly polymeric systems, remain essential for propulsion and hypersonic protection, with their thermal response commonly assessed by differential scanning calorimetry (DSC), thermogravimetric analysis (TGA), thermomechanical analysis (TMA), and differential thermal analysis (DTA), while oxy-acetylene torch (OAT) testing and simulated solid rocket motor (SRM) tests more accurately reproduce hyperthermal environments [4].

TPS development must prioritize mass-efficient materials, accurate modeling and simulation, and advanced sensors to ensure optimal performance, reduce mission risks, and enable in-space damage detection and repair [5]. Concerning advancements, the development of high-strength hollow microspheres (HMs) has made possible low-damage, low-density TPS materials with enhanced ablation resistance, addressing HM breakage during extrusion and achieving greater density reduction than previously reported, thereby improving SRM performance [6]. To improve weight saving, dual-layer ablatives enhance TPS performance via customizable layering, with uniform composites as the foundation, supporting missions like Dragonfly [7]. Emerging commercial and NASA missions demand low-cost, rapidly producible, and environmentally friendly ablative TPS, prompting the development of PICA-Flex, a hybrid blanket from the MERINO family intermingling carbon and phenolic fibers [8]. Moreover, boron trioxide (B_2_O_3_)-reinforced polymer matrix composites (PMCs) enhanced char-layer antioxidation, reducing ablation by 48% and increasing residue and liquid-film content, with highly oriented pyrolytic graphite (HOPG) formation improving TPS performance in varied SRM environments [9].

Thermal protection system material development is validated via testing, such as thermal–fluid–ablation models showing that local thermal non-equilibria and radiation strongly affect responses based on pore sizes greater than 1 micrometer and porosity [10], or by computational fluid dynamics (CFD), which is used to simulate the complex flow of hot gases and predict heat transfer during atmospheric entry [11,12]. A flame exposure apparatus, set up according to the American Society for Testing and Materials (ASTM) E 285-80 standard and validated via CFD simulations of oxygen–acetylene combustion, accurately predicted heat flux and surface temperatures, providing a benchmark for TPS material testing [13]. Two additional TPS classes combining a carbon fiber reinforced polymer (CFRP) layer with phenolic-based ablatives, cork, or carbon felt reinforced with 1–2 weight percentage nano-silicon carbide (nSiC) and joined by a ceramic adhesive, were oxy-butane (OTB)-tested. These tests showed that carbon felt reinforcement and nSiC doping improved thermal conductivity, reduced mass loss, and limited erosion via energy dissipation and silicon carbide (SiC) to silicon dioxide (SiO_2_) oxidation, confirming high thermal resistance for aerospace applications [14].

Five high-temperature thermoplastics—two polyetherimides (PEIs), two polyether ether ketones (PEEKs), and one polyether ketone ketone (PEKK)—were fabricated by fused filament fabrication (FFF) three-dimensional (3D) printing and characterized for ablation and thermal performance. Among them, PEKK exhibited the highest char yield (64 weight percent) and best residual mass under 100 W/cm^2^ OAT and inductively coupled plasma (ICP) tests, while the PEIs exhibited the lowest flammability measured by microscale combustion calorimetry (MCC, heat release 408 J/g-K) and the PEEKs showed the highest thermal decomposition temperatures, confirming material-dependent intumescent behavior and porous char formation under rapid heating [15]. The PEKK composites reinforced with 10 weight percentage carbon nanotubes (CNTs), carbon fibers (CFs), and glass fibers (GFs) were fabricated by FFF 3D printing and tested under TGA and OTB exposure. CNT–PEKK showed the highest char yield (69 weight percent), lowest mass loss, minimal swelling, and best thermal performance, while CFs and GFs increased effusivity but also swelling [16]. Moreover, polyacrylonitrile carbon fiber (PAN-CF) carbon/phenolic composites (CPCs) were OAT-tested for in-plane and out-of-plane thermal diffusivity, showing agreement with reference materials MX-4926 and FM-5014 under high heating rates [17].

For the development of the manufacturing process, additive manufacturing of concrete for TPS applications employed a traveling salesman problem (TSP)-based continuous print path with contour offset and sharp-turn removal, improving print quality, structural integrity, and reducing anisotropic strength, as validated through experimental and numerical evaluation of planar and topology-optimized designs [18]. Additive manufacturing of ablative TPS was also demonstrated using a low-cost five-axis extrusion printer and a high-viscosity, 3D-printable composite, producing char yields comparable to legacy materials and enabling in situ deposition, scalable production, and reduced tile fabrication and assembly costs [19].

This study builds upon recent advancements in additive manufacturing of ablative thermal protection systems (TPSs), demonstrating that ceramic-coated and hybrid or continuous fiber-reinforced composites improved ablation resistance, while the incorporation of internal air chambers enhanced lightweight performance. The novelty of incorporating 3D-printed air chamber architectures effectively reduces thermal conductivity and convective heat transfer, enabling scalable, in situ fabrication while maintaining high char yield and structural integrity. These innovations distinguish the present work from previous studies and mark a significant advancement in the next generations of TPS design, bridging material innovation and advanced manufacturing strategies.

## 2. Materials and Methods

### 2.1. Design Concept and Hypothesis

A central element of the present investigation is the development of a new thermal protection system (TPS) architecture integrating internal air chambers as a means of enhancing thermal management under ablative conditions. Unlike conventional solid infill geometries, the proposed design incorporates three individual distributed air cavities, separated by walls of controlled thicknesses, thereby creating internal barriers to heat conduction and convection. The basic principle underlying this approach is the introduction of discrete airspaces within the bulk material, which are expected to act as thermal insulators while simultaneously contributing to overall weight reduction.

**Figure 1:**
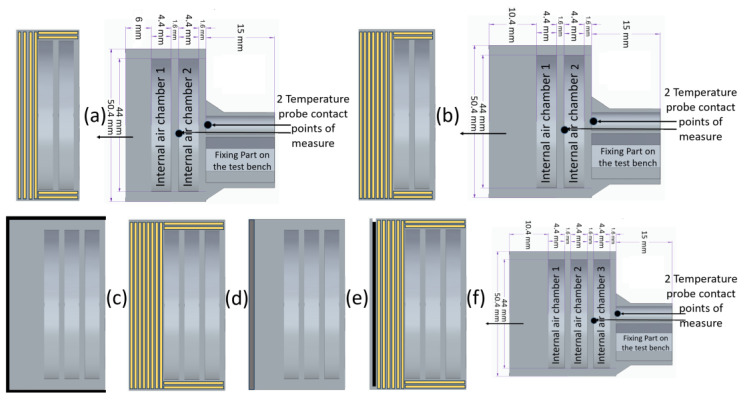
Probe configurations differentiated by air chamber number and hybridization: (**a**) Configuration 1, (**b**) Configuration 2, (**c**) Configuration 3, (**d**) Configurations 4–5, and (**e**) Configurations 6–7. All three-chamber designs are detailed in (**f**).

Design sketches of the developed configurations (Figure 1) and the data presented in Table 1 illustrate the proposed architectures. Figure 1 presents the probe configurations, differentiated by air chamber number and hybridization. Configuration 1 is shown in (a), Configuration 2 in (b), and Configurations 3, 4, 5, 6, and 7, each comprising three air chambers as detailed in (f), are illustrated in (c), (d), and (e), respectively. The sketches represent the internal architectures of the probes rather than the actual reinforcements or hybridizations. Each configuration was fabricated using additive manufacturing using a fire-retardant thermoplastic material, with certain specimens further reinforced by continuous carbon or glass fibers. Beyond continuous reinforcement, variations in wall thickness were introduced, alongside the application of ceramic thermal coatings or CFRP hybridizations, to assess the combined influence of geometry, structural design, and material adjustments on thermal stability and ablative performance.

Table 1 presents the configurations developed for the new TPS system prototypes, as mentioned above. The preliminary architectural concept, originating from a patent request that was filed prior to the present work, in relation with the new internal air chamber concept, is a heat shield obtained through additive manufacturing with a low weight reference and air/vacuum chambers intended for space applications, featuring air chambers (two or three) equipped with openings that allow exchange with the external environment. In vacuum conditions, both conductive and convective heat transfer are suppressed, rendering radiation the dominant mechanism for thermal energy dissipation. Although air’s thermal conductivity slightly increases with temperature, it decreases under low-pressure conditions. Onyx FR-V0 constitutes the flame-retardant variant of Onyx, a nylon-based (polyamide 6) composite containing 10–20 vol% short carbon fibers [20]. The material exhibits a density of 1.2 g/cm^3^ and a heat deflection temperature of 145 °C. It demonstrates advantageous mechanical performance, high thermal stability, and resistance to ultraviolet radiation and chemical agents. When reinforced with continuous carbon fibers, Onyx FR-V0 achieves mechanical properties comparable to those of 6061-T6 aluminum, which is attributed to fiber lengths predominantly aligned with the deposition direction, ranging from 7.035 to 44.58 μm [21].

Based on the architectural concept, the selected material configurations, and the prototypes, it is hypothesized that the integration of internal air chambers will (i) reduce effective thermal conductivity by limiting direct heat transfer pathways; (ii) dissipate incident heat flux more effectively, thereby lowering backside temperatures; (iii) decrease overall system mass relative to fully infilled structures; and (iv) in case of reinforced configurations or hybridization, exploit the anisotropic thermal conductivity of the fibers to distribute heat preferentially along aligned directions, thus mitigating localized degradation. The hybrid and continuous fiber-reinforced composites provided superior ablation resistance, further supporting this approach. Collectively, these mechanisms and structures are expected to improve both thermal stability and structural integrity under extreme oxy-acetylene torch exposure, validating the viability of the proposed architecture for next-generation 3D-printed ablative TPSs.

### 2.2. Materials, Manufacturing, and Methods

The fabrication of the ablative TPS specimens was carried out using fused deposition modeling (FDM) additive manufacturing, produced from Onyx FR-V0. Configurations 3 and 6 were produced on a Prusa XL (Prusa Research a.s., Prague, Czech Republic) printer with 1.75 mm filament, while Configurations 1, 2, 4, 5, and 7, also based on Onyx FR-V0 but reinforced with continuous carbon or glass fibers, were manufactured on a Markforged X7 (Markforged X7, Waltham, MA, USA), employing 310 µm fiber filaments. Printing on the Prusa XL was performed at a nozzle temperature of 290 °C, a bed set heated to 110 °C, and with a layer thickness of 0.2 mm. In contrast, the Markforged X7 builds were carried out at a nozzle temperature of 275 °C, with an unheated platform, a layer thickness of 100 µm, a single shell, a nozzle diameter of 0.4 mm, and full (100%) infill. Configurations 3 and 6 adopted a two-shell wall design, whereas Configurations 1, 2, 4, 5, and 7 employed a single-shell wall structure.

All configurations incorporated a full-infill FDM build using Onyx FR-V0 as the base material and included either a dual-chamber or tri-chamber internal air cavity design with radial walls 3.2 mm in thickness. One specific category of specimens (Configuration 1) was distinguished by a reduced frontal wall thickness of 6 mm, while all remaining designs employed a 10.4 mm frontal wall. Given that the carbonaceous layer formed by the locally reinforced matrix is inherently prone to erosion, continuous fiber reinforcement with either carbon or glass fibers was implemented in nearly all configurations. These reinforcements provide an additional heat-absorbing function through endothermic melting and evaporation, while simultaneously limiting mechanical erosion during high-temperature exposure. For all reinforced configurations (except 3 and 6), the frontal wall further differed by the inclusion of concentric continuous fiber reinforcement rings, with the number ranging from four to eight and consisting of either carbon or glass fibers. In the case of radial walls, the number of reinforcement rings remained constant, with all reinforced specimens containing precisely two concentric fiber rings regardless of fiber type.

Configurations 3 and 7 were additionally hybridized by integrating carbon fiber-reinforced polymer CFRP elements on the flame-exposed surface. In the case of Configuration 3, an additional CFRP (Hexply M49/42%/200T2X2/CHS-3K) layer was applied to all the external surfaces of the sample (front and radial walls) and cured for 60 min at 120 °C under a vacuum pressure of −0.5 bar. In Configuration 7, a cured CFRP laminate disk (Hexply M49/42%/200T2X2/CHS-3K) was embedded within a 2 mm slot at the exposure interface to enhance thermal shielding. By contrast, Configuration 6 incorporated a surface modification consisting of a 3 mm layer of aluminum oxide (Al_2_O_3_) powder with epoxy resin. The incorporated filler was a hard brown alumina mixture (>95.5% Al_2_O_3_) characterized by a Mohs hardness of 9.0, a density of 3.9 g/cm^3^, a melting point of 2200 °C, and a particle size corresponding to an 80-mesh grit (approximately 110 µm).

### 2.3. Experimental Protocols

**Figure 2:**
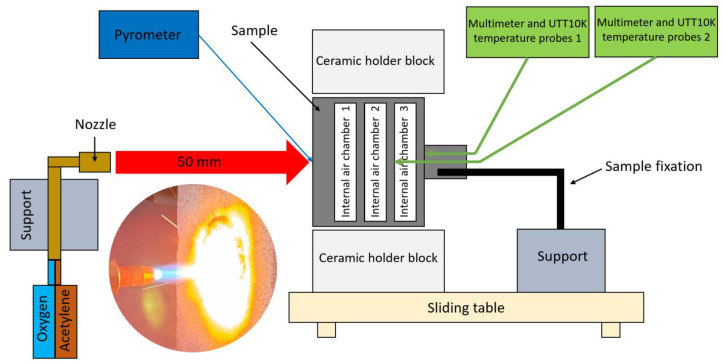
Oxy-acetylene torch test bench (OTB) for ablation assessment [22].

High-temperature oxidation testing was performed using a purpose-built oxy-acetylene torch rig, with a schematic of the facility shown below in Figure 2. A welding nozzle (LS14 model, inner No. 4 nozzle, RHONA GCE group, Steinhausen, Switzerland) supplied a mixture of oxygen and acetylene to produce an oxidizing flame with a peak temperature of 1600 ± 50 °C. The samples, each 50 mm in diameter with geometric features detailed above, in Figure 1, were placed in a ceramic holder featuring a 51 mm diameter circular opening to ensure accurate positioning. The holder was mounted within a rigid support structure on a mobile roller plate equipped with a locking mechanism, which allowed for precise and repeatable placement of each sample relative to the flame.

For testing, the mobile roller plate was manually advanced so that the flame was centered on the front surface of the sample, maintaining a nozzle-to-sample distance of 50 mm to achieve the desired heating profile. Two samples were tested for each configuration: one exposed to the flame for 60 s and the other for 90 s. An exception was made for sample 6.2, which was exposed to the flame for only 60 s rather than the intended 90 s. This reduction was necessary because the degradation rate exceeded expectations, given the extreme testing conditions. After exposure, all samples were removed from the flame field by retracting the roller plate, allowing for natural cooling. Throughout the test campaign, the flame was kept continuously lit to ensure the consistency and reproducibility of the testing conditions.

Temperature measurements were conducted at both the front and rear of the samples. The peak temperature at the center of the front surface was recorded using an infrared pyrometer (Sonel DIT-500 from SONEL S.A., Swidnica, Poland). Internal temperatures on the back face were measured at two points on the rear walls of the second (T1f) and third air chambers (T2f) (viewed from the front, Figure 2) using UTT10K UNI-T temperature measure probes, which have a range up to 260 °C and ±0.75% accuracy. These probes were connected to two digital multimeters (UT131C from UNI-Trend Technology Co., Ltd., Shenzhen, China, and SMA 19 Somogyi Elektronic Kft., Gyor, Hungary) for data acquisition.

## 3. Results and Discussions

Following the Oxy-acetylene Test Bed (OTB) ablation tests, a comprehensive post-test analysis was conducted, which included visual inspection, morphological characterization, and metrological measurements of both weight and thickness. Weight measurements were performed with a Kern PLJ 510-3M (KERN & SOHN GmbH, Balingen, Germany) analytical balance, which provided a precision of ±0.001 g. Thickness measurements were taken using a Mitutoyo CD-P15P (Mitutoyo Corporation, Kawasaki, Japan) digital vernier caliper with a measuring range of 0–150 mm and an accuracy of 0.01 mm. These systematic measurements provided quantitative data for evaluating the material’s performance and the extent of degradation.

Table 2 depicts images of the specimens after oxy-acetylene torch (OTB) ablation testing. Some configurations developed a carbon-rich char layer, interspersed with dark-brown regions indicative of localized oxidation. Unlike conventional thermoset-based ablators, which form thick and cohesive protective char layers, the thermoplastic systems investigated here produced thinner and more fragile char. This behavior is attributed to the decomposition pathways of thermoplastics, which involve rapid pyrolysis and volatile release at relatively low temperatures, thereby limiting the accumulation of a dense insulating layer.

The hybridized configurations exhibited differentiated surface responses under ablation testing compared to the other configurations. Specimens modified with an external reinforcement CFRP layer (Hexply M49/42%/200T2X2/CHS-3K) preserved comparatively cleaner surfaces with less charring after exposure. Configuration 7, which incorporated a cured CFRP laminate disk embedded within a 2 mm slot at the flame-facing interface, demonstrated a similar protective effect, maintaining surface integrity aside from localized damage at the flame impingement zone. In contrast, Configuration 6, which integrated a 3 mm alumina (Al_2_O_3_) surface layer, exhibited accelerated degradation, with significant surface penetration and reduced structural preservation relative to the CFRP-modified counterparts. Across all hybridized specimens, material loss was concentrated at the region corresponding to the flame diameter, whereas peripheral areas remained largely unaffected. The results demonstrate the superior shielding capacity of CFRP-based modifications, although the internal structure of air chambers was still affected, while the alumina-filled configuration proved limited due to rapid erosion of the alumina–epoxy layer under high thermal flux and reduced stand-off distance.

Post-test analysis revealed that all Onyx FR-V0 specimens, whether reinforced or hybridized, developed a carbon-rich char layer after 60 s and 90 s of oxy-acetylene exposure. This layer acted as a transient thermal barrier by insulating and re-radiating heat, and dissipating energy through progressive erosion. Nonetheless, local detachment was observed in some specimens, while others exhibited stronger adhesion, likely due to liquid phase formation at elevated temperatures and re-solidification upon cooling. Reinforced specimens outperformed hybridized ones, forming denser, more stable char layers. This behavior is attributed to the oxidative degradation of Onyx FR-V0, where oxygen-induced carbonyl groups enhanced char cohesion in fiber-reinforced architectures.

According to Table 3, Specimens 1 and 6 showed the most extensive damage under both exposure times, with structural deformation indicating failure. Configuration 1 experienced significant deformation leading to critical damage, primarily due to the thinner frontal wall and the limited reinforcement provided by only four continuous carbon fiber-reinforced rings in the frontal area.

Nevertheless, the char layers of both specimens were porous and fragile, detaching even after 60 s and leading to structural collapse. In contrast, other reinforced and hybridized specimens retained adherent char layers, preserving integrity.

For Specimen 2.1 (10.4 mm frontal wall), the cross-section after testing showed ablation and thermo-oxidative degradation, with a rough surface, material loss, and exposed carbon fiber bundles due to matrix pyrolysis. Significant porosity was present, originating from both FFF process-induced voids and thermal-degradation gases. The longer exposure of Specimen 2.2 intensified ablation, reducing wall thickness and producing deeper erosion with extensive pits. The thermal front penetrated further, compromising the thin layer between air chambers and weakening insulation capacity.

Across hybridized configurations, prolonged exposure consistently worsened degradation. Specimens 3.1 and 3.2 illustrate this trend; 3.1 (60 s) retained intact air chambers under a protective char layer with localized porosity, while 3.2 (90 s) exhibited accelerated ablation, reduced wall thickness, and thermal front propagation that compromised internal layers. Similarly, Specimens 7.1 and 7.2 shifted from controlled degradation to severe plastic deformation; 7.1 (60 s) pre-served geometry via a char layer formed by CCF and CFRP reinforcements, whereas 7.2 (90 s) showed a collapse of air chambers, extensive deformation, and dislodgement of CCF reinforcements, indicating a loss of structural capacity.

Specimens 4.1 and 4.2 further emphasize exposure duration effects; 4.1 (60 s) showed early degradation with moderate roughness and incipient char while maintaining integrity, whereas 4.2 (90 s) displayed severe material loss, wall thinning, deeper erosion, and the degradation of internal structures. Similarly, Specimens 5.1 and 5.2 highlight this progression; 5.1 (60 s) remained structurally sound with only initial surface degradation, while 5.2 (90 s) underwent significant ablative thinning, extensive porosity, and thermal front penetration into the core. Furthermore, in contrast to Probe 4.1, which was reinforced with continuous carbon fiber, the degradation observed in Probe 5.2, which was reinforced with continuous glass fiber has advanced to such an extent that the underlying onyx layer becomes distinctly visible.

When comparing Configurations 1 and 2, integrating only a two-air-chamber design, it is evident that the structural integrity of Specimens 1.1 and 1.2 was significantly compromised. Although, analyzing exclusively the surface degradation after 60 s of exposure, Configuration 2 seems more affected than Configuration 1; when examining the cross-sections, it is evident that Configuration 1 is structurally damaged. Likewise, when increasing the exposure time to 90 s, Configuration 1 (Sample 1.2) is clearly more affected by the damage being localized in a narrow region in the center of the exposed surface, the area directly subjected to the flame, and this is obvious when investigating both surface and section views. Specimens 1.1 and 1.2 exhibited major deformation after 60 s, followed by the collapse of the frontal wall and the overall structure after 90 s. This behavior is primarily attributed to the thinner frontal wall (6 mm) and the presence of only four CCF-F (continuous carbon fiber-reinforced) rings in the frontal wall area. In contrast, Configuration 2 featured a thicker frontal wall (10.4 mm) and eight CCF-F reinforcement rings, which provided greater resistance. However, it is important to note that the CCF-F-reinforced radial wall area in both configurations maintained sufficient structural integrity during the first 60 s of exposure. Thus, the degradation began at the center of the exposed surface, in the area directly subjected to the flame, eventually leading to structural failure after 90 s. Consequentially, a two-air chamber architecture will ensure a decrease in the overall system mass and can provide the necessary thermal conductivity reduction by limiting direct heat transfer pathways but only if integrated in a continuous reinforced structure in both frontal and radial wall areas to benefit from the anisotropic thermal conductivity of the fibers to distribute heat preferentially along aligned directions, thus mitigating localized degradation, as in the case of Configuration 2.

Configuration 2 showed the best performance, with thicker frontal walls and more CCF-F reinforcement preserving structural integrity under thermal load. Configuration 1 failed after 90 s, while hybridized three-air-chamber designs degraded progressively, with partial char protection at 60 s but significant ablation at 90 s. In all probes from Table 3, internal air chamber structures remained intact, though minor deformations occurred, underscoring the role of wall thickness and continuous reinforcement.

Following the removal of the char layer by brushing the surface, a detailed analysis of the thickness change rate, thickness, and mass loss was conducted to quantify the material’s ablative performance. Thickness change rate is a key indicator of thermal protection duration, and it was calculated by dividing the thickness loss by the exposure time. As in Allcorn et al. [23], the thickness after testing was defined as the thickness of the post-test virgin material. Consequently, the recession value quantifies the progression of the reaction front into the virgin material, excluding the residual char layer. As is desired, low recession values signify superior thermal protection. 

Table 4 illustrates the results of the thickness change rates and provides cross-section views of all tested specimens.

Post-test analysis of the probes revealed the thickness change rate, which is a key metric for ablative performance, directly correlated with the extent of material degradation. The most significant thickness change rates were observed in Probes 1.1, 1.2, and 6.1, which experienced failure. In contrast, all other configurations demonstrated a more favorable performance, and with Probes 5.1 and 5.2 exhibiting the lowest thickness change rates, indicating superior thermal protection and structural integrity. The positive thickness change rates observed for Probes 2.2 and 4.1 are indicative of material buildup on the surface. This phenomenon is commonly attributed to the formation and densification of an expanded char layer, which projects beyond the original specimen plane.

The data confirms a direct relationship between increased thermal exposure duration and the extent of material loss. For instance, the transition from a 60 s to a 90 s exposure in most configurations led to a significant acceleration in the rate of ablative material removal. The most severe degradation was characterized by a profound increase in frontal wall thinning, deeper surface erosion, and the propagation of the thermal front into the internal structure, which in some cases compromised the internal structures of the air chambers. This highlights the critical role of exposure duration in determining the long-term effectiveness of the material’s thermal protection system.

**Figure 3:**
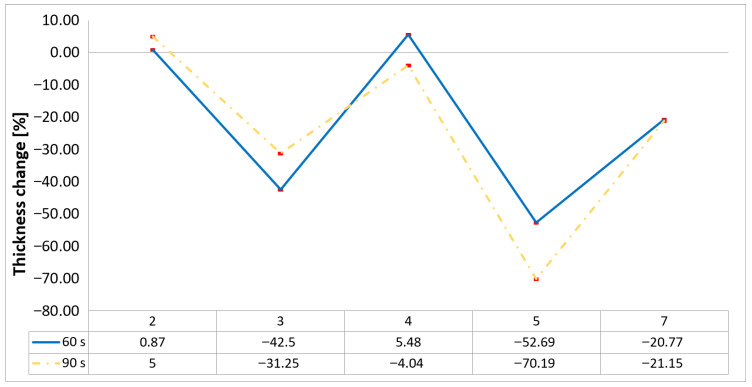
Thickness changes chart of tested configurations following char layer elimination.

Following the analysis of thickness change rates, a more direct quantification of ablative performance is the total thickness loss. This metric, illustrated in the subsequent chart (Figure 3), shows the absolute reduction in material thickness for each tested specimen. Unlike the thickness change rate, which is an averaged value, thickness loss directly represents the total physical degradation, offering a clear visual and quantitative comparison of how effectively each configuration resisted the thermal load and protected its structural integrity.

The analysis of thickness change data reveals significant variations in the ablative performance of the tested probes. Probes 3 and 5 experienced the most substantial thickness change, with Probe 5 losing over 50% of its thickness in the 60 s test and more than 70% in the 90 s test. This indicates considerable material erosion and a failure to maintain structural integrity under extended thermal load. In contrast, Probes 2 and 4 demonstrated a markedly different trend. Probe 2 showed minimal thickness change across both exposure times, while Probe 4 exhibited a positive thickness change after 60 s. The latter suggests a swelling effect attributed to the formation of a protective char layer, which indicates superior thermal resistance and effective mitigation of material recession.

**Figure 4:**
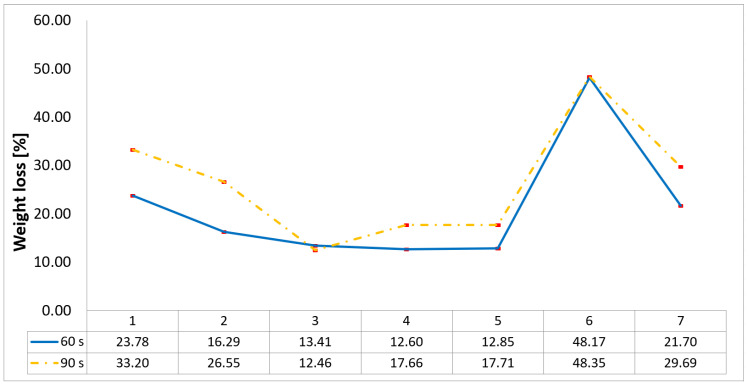
Weight loss of 3D-printed composite TPS configurations under OTB ablation testing.

The weight loss data (Figure 4) generally corroborates the trends observed in thickness change. Probes 1 and 6 displayed the highest weight loss percentages, signifying extensive mass ablation and material consumption, consistent with their overall structural failure. However, a notable discrepancy was observed in Probe 5, which, despite its significant thickness loss, showed one of the lowest weight loss percentages, particularly after the 60 s exposure. This apparent contradiction suggests a degradation mechanism distinct from simple mass ablation, where a portion of the material is sacrificed to form a lightweight, thermally insulating char layer.

Configurations 4, 5, and 3, all integrating a three-air-chamber design, showed the lower weight loss percentages, followed closely by 2 and 7. This trend is potentially due to CFRP protective layer that, however, once penetrated, left the unreinforced structure exposed due to the high thermal flux leading to accelerated degradation, as in the case of Configuration 3. Configurations 4 and 5 join the same structural design, the difference consisting in the continuous reinforcement phase-type carbon and, respectively, glass. Their lower weight loss percentages indicate an ablation protection provided by reinforcement phase primarily in the frontal and then in the radial regions, while the anisotropy of these composite structures promoted heat dissipation along fiber directions, limiting thermal penetration into the bulk and preventing excessive surface heating. Configuration 2, with two air chambers integrated into its internal structure, exhibits slightly higher weight loss percentages when compared to configurations discussed above (3, 4, and 5), which feature three-air-chamber designs. This indicated that a two-air-chamber design can sustain high thermal flux levels, if its TPS structure integrates both a thick frontal wall and high continuous reinforcement (eight carbon rings), which provide greater resistance. By contrast, Configuration 7, with a three-air-chamber design, displays significantly higher weight loss percentages when compared to the other tested configurations (except for Configuration 6), and this is due in all likelihood to the flame’s lack of centration and accelerated degradation of the radial wall, with the surface protective CFRP laminate being partially removed from the surface.

**Figure 5:**
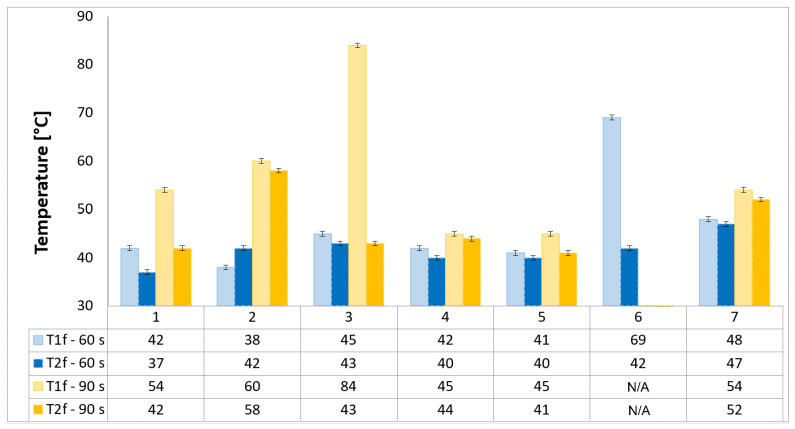
Temperature evolution of tested OTB ablation configurations as a function of exposure time.

The temperature chart (Figure 5) provides critical insights into the probes’ thermal insulation capabilities. From this, it can be generally observed that the temperature values measured at both locations remain significantly lower than the maximum allowable TPS back-face temperature of 180 °C, thereby ensuring that the spacecraft and its components remained within design safety margins.

Lower temperatures on the back face of the probe (T1f and T2f) are desirable, as they indicate effective thermal protection. Probe 6 shows an extremely high temperature reading on its back face at the 60 s mark (69 °C). Probe 3 demonstrates a significant increase in temperature at the back face with this, for the 90 s exposure, jumping to 84 °C. This suggests that while it may perform well in shorter tests, its long-term insulating capacity is compromised.

Conversely, Probes 4, 5, and 7 maintain relatively low back-face temperatures even during the 90 s exposure, all staying below 45 °C. This indicates that these configurations are highly effective at preventing heat from propagating through the material, despite varying degrees of thickness and weight loss. Probe 5, in particular, shows remarkable thermal insulation performance, maintaining a low back-face temperature despite its significant thickness loss. This suggests that its degradation mechanism results in a highly insulative, porous char layer.

Configurations 2, 4, and 5 are the most promising TPS designs, as their continuous reinforcement in their frontal and radial regions ensures ablation resistance and reduced back-face temperatures through anisotropic in-plane heat conduction; among them, Configurations 4 and 5, identical in design but reinforced with carbon and glass, respectively, exhibit the lowest weight loss and thermal loads, while Configuration 2, with thick reinforced walls, further demonstrates ablation resistance and highlights the potential of a two-air-chamber architecture to decrease system mass and enhance insulation, provided reinforcement continuity enables anisotropic redistribution of thermal loads and mitigates localized degradation.

## 4. Design Optimization Pathways

The post-test analysis of Onyx FR-V0-reinforced and -hybridized specimens provides clear guidance for optimizing thermal protection system (TPS) architectures. Reinforcement strategy emerged as a primary determinant of performance: continuous carbon fiber reinforcement consistently promoted the formation of dense, cohesive char layers, whereas hybridized configurations with combined CCF and CFRP elements exhibited localized char detachment under extended thermal exposure. Consequently, prioritizing continuous fiber reinforcement in critical frontal regions enhances char cohesion and mitigates premature surface failure.

Combining the benefits from Configurations 2 and 3, a two-air-chamber internal design is viable for TPSs when the frontal and radial walls are reinforced with continuous carbon or glass fibers.

Frontal wall thickness and internal structural design were also shown to strongly influence ablative behavior. Probes with thicker frontal walls and well-protected air buffers, such as 2.1, 4.1, and 5.1, maintained structural integrity and limited back-face heating, whereas thinner walls and poorly supported hybridized layers (e.g., Probes 1 and 6) suffered accelerated ablation and internal degradation. Optimizing wall thickness while preserving lightweight characteristics is therefore essential to balance thermal resistance and material efficiency.

Additionally, material selection at the fiber–matrix interface must account for oxidative degradation and char formation. The enhanced char cohesion observed in Onyx FR-V0 reinforced with carbon fibers indicates that tailoring fiber–matrix chemistry to promote carbonyl group formation and controlled porosity can maximize thermal insulation and energy dissipation. Design strategies should, therefore, integrate reinforcement continuity, interface chemistry, and structural geometry to achieve superior ablative performance under both short- and long-duration thermal loads.

## 5. Implications for Aerospace Applications

The dense, cohesive char layers observed in reinforced configurations suggest applicability to short-duration, high-intensity thermal events such as atmospheric re-entry of spacecraft, ascent through hypersonic flight regimes, and localized engine or nozzle heating. Probes 4, 5, and 7, which maintained low back-face temperatures despite significant thickness loss, demonstrate the potential for preserving structural integrity in critical TPS components while minimizing weight penalties.

In contrast, Probes 1 and 6, exhibiting porous char and rapid frontal wall erosion, illustrate the risk of failure under extended or repeated thermal loads, relevant to missions with prolonged exposure to aerodynamic heating, such as long-duration hypersonic cruise or sustained high-speed flight in low Earth orbit (LEO). These findings underscore the importance of continuous fiber reinforcement, optimized frontal wall thickness, and controlled fiber–matrix interactions to enhance char cohesion, energy dissipation, and thermal insulation.

## 6. Conclusions

The Onyx FR-V0-based TPS prototypes were successfully tested under controlled oxy-acetylene flame exposure, demonstrating their capability to withstand extreme thermal conditions.

The study shows that carbon-rich char formation is the primary mechanism of thermal protection, providing insulation, energy dissipation, and heat re-radiation.

Reinforced configurations consistently produced denser, more cohesive char, whereas hybrid variants exhibited higher porosity and localized detachment, resulting in lower performance.

Specimens with continuous frontal and radial reinforcement, particularly Configurations 2, 4, and 5, showed superior ablative resistance, reduced back-face temperatures, and minimal mass loss, highlighting the importance of reinforcement strategy, wall thickness, and fiber–matrix interactions.

Two-air-chamber architectures effectively reduce system mass while maintaining comparable thermal insulation, provided that continuous reinforcement promotes preferential in-plane heat conduction and limits localized degradation.

Overall, these findings identify key design parameters for the future optimization of Onyx FR-V0-based TPSs, enabling maximized char cohesion, controlled porosity, and sustained thermal protection for advanced aerospace applications.

## 7. Patents

A national patent request was filled prior to the present work in relation with to the new internal air chamber concept of a heat shield obtained through additive manufacturing with a low weight reference and air/vacuum chambers intended for space applications: Teodor-Adrian Badea, Alexa Crisan, and Raluca Maier, reference no. A/00073 OSIM: 26 February 2025.

## Figures and Tables

**Table 1:** Description and nomenclature of novel, additively manufactured composite ablative thermal protection system (TPS) configurations, which integrate a new internal air chamber architecture, hybridization, and a ceramic surface coating ^1^.

Material	Configuration Code ^1^	Sample Code/Mass *			
		Sample (60 s)	Mass [g] *	Sample (90 s)	Mass [g] *
Onyx FR-V0	CCF-F (4 rings)/CCF-R/AC (2)/TW/ThFW/	1.1	28.163 ± 0.002	1.2	29.118 ± 0.002
	CCF-F (8 rings)/CCF-R/AC (2)/TW/TFW	2.1	36.254 ± 0.002	2.2	39.176 ± 0.002
	CFRP-1 PLY/AC (3)/TW/TFW	3.1	43.985 ± 0.002	3.2	43.997 ± 0.002
	CCF-F (8 rings)/CCF-R/AC (3)/TW/TFW/	4.1	46.428 ± 0.002	4.2	45.447 ± 0.002
	CGF-F (8 rings)/CGF-R/AC (3)/TW/TFW	5.1	50.71 ± 0.002	5.2	52.318 ± 0.002
	Al_2_O_3_-SC/AC (3)/TW/TFW	6.1	36.204 ± 0.002	6.2	36.034 ± 0.002
	CFRP-disk/CCF-F (6 rings)/CCF-R/AC (3)/TW/TFW	7.1	43.013 ± 0.002	7.2	43.139 ± 0.002

^1^ (Onyx FR-V0 reinforced with 10% chopped carbon fiber); CCF-F—Continuous Carbon Fiber-reinforced Frontal wall area; CCF-R—Continuous Carbon Fiber-reinforced Radial wall area; AC—Air Chambers (two or three); TW—Thick radial Wall of 3.2 mm; ThFW—Thin Frontal Wall of 6 mm; TFW—Thick Frontal Wall of 10.4 mm; CFRP-PLY-1 layer of CFRP entirely covering the samples cured for 60 min at 120 °C under −0.5 bar; CGF-F—Continuous Glass fiber-reinforced frontal wall area; CGF-R—Continuous Glass fiber-reinforced Radial wall area; Al_2_O_3_-SC—Al_2_O_3_ powder surface coating of 3 mm with epoxy resin; CFRP-disk—mechanically integrated CFRP disk laminate; * mass weights were determined prior to tests (excluding test rig fixation parts).

**Table 2:** Samples after OTB ablation testing: configuration/exposure time, frontal view, side view, and frontal view after brushing following char removal.

60 s		90 s	
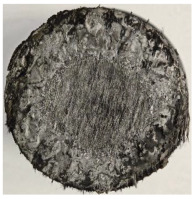	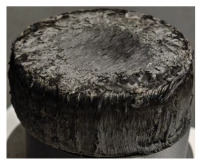	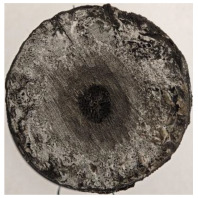	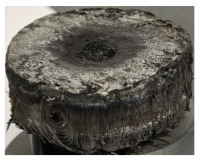
1.1 frontal view	1.1 side view	1.2 frontal view	1.2 side view
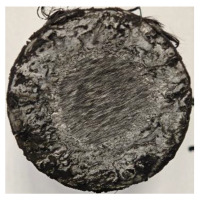	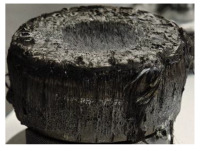	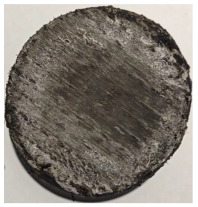	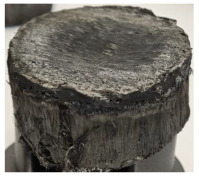
2.1 frontal view	2.1 side view	2.2 frontal view	2.2 side view
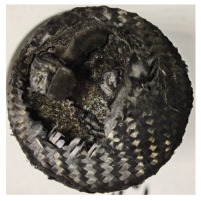	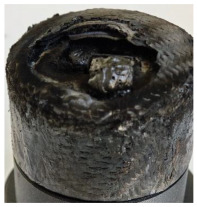	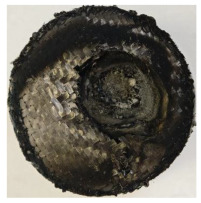	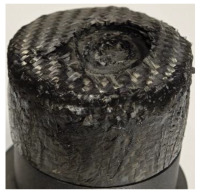
3.1 frontal view	3.1 side view	3.2 frontal view	3.2 side view
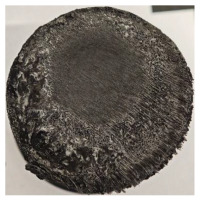	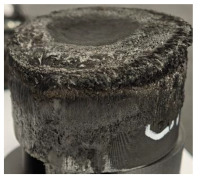	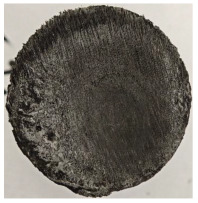	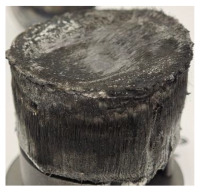
4.1 frontal view	4.1 side view	4.2 frontal view	4.2 side view
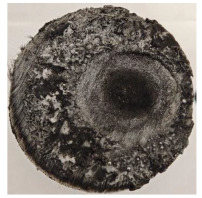	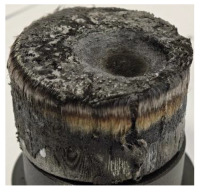	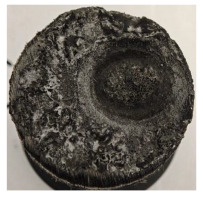	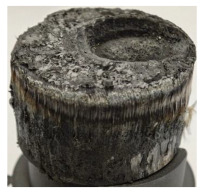
5.1 frontal view	5.1 side view	5.2 frontal view	5.2 side view
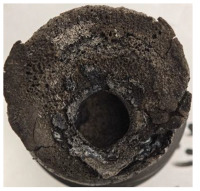	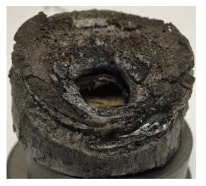	N/A	N/A
6.1 frontal view	6.1 side view	6.2 frontal view	6.2 side view
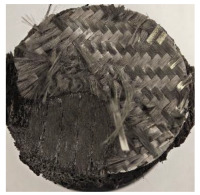	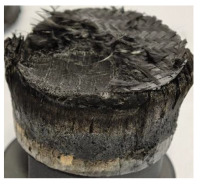	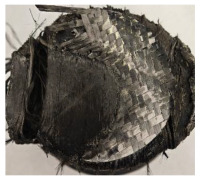	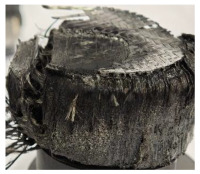
7.1 frontal view	7.1 side view	7.2 frontal view	7.2 side view

**Table 3:** Post-OTB ablation testing: visual assessment of surface degradation and porosity in key probes.

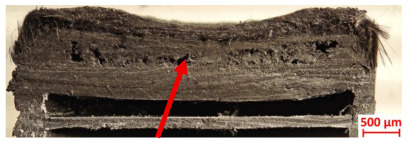	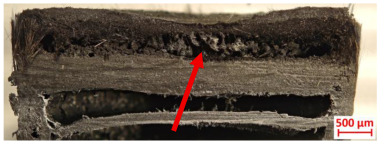
2.1	2.2
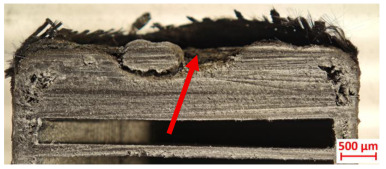	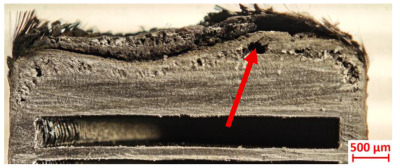
3.1	3.2
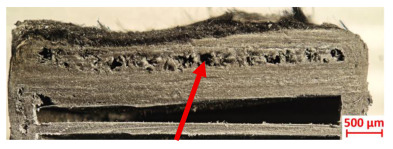	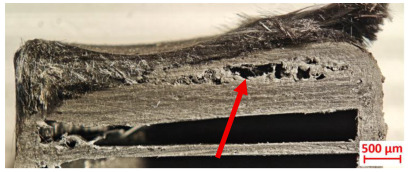
4.1	4.2
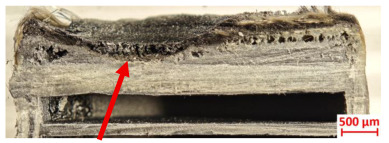	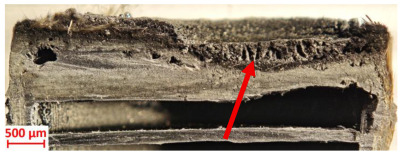
5.1	5.2
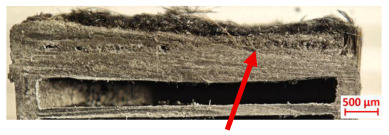	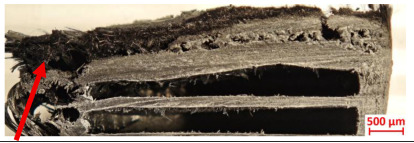
7.1	7.2

**Table 4:** Cross-section visual assessment and thickness change rates of all the tested probes following OTB ablation testing.

60 s	Thickness Change Rate (mm/s)	90 s	Thickness Change Rate (mm/s)
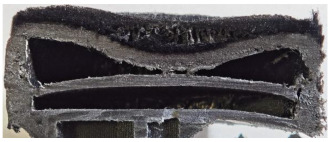	N/A	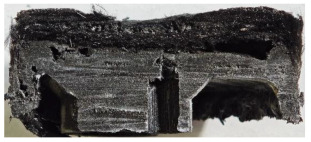	N/A
1.1		1.2	
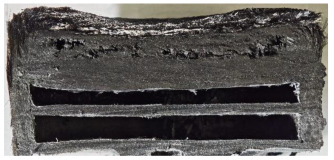	−0.002 ± 0.0002 mm/s	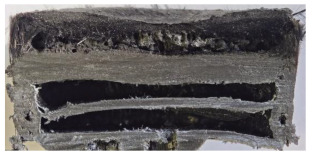	+0.009 ± 0.0002 mm/s
2.1		2.2	
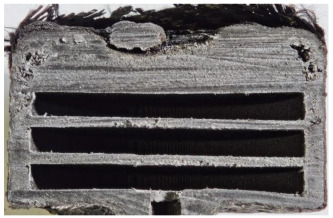	−0.074 ± 0.0002 mm/s	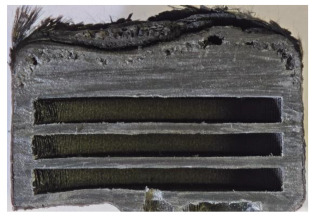	−0.054 ± 0.0002 mm/s
3.1		3.2	
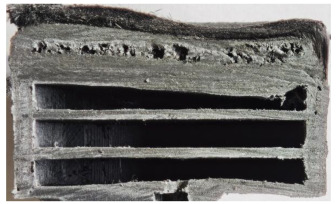	+0.010 ± 0.0002 mm/s	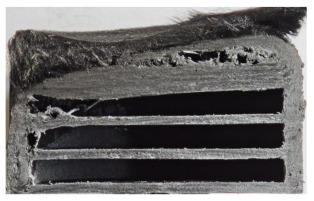	−0.007 ± 0.0002 mm/s
4.1		4.2	
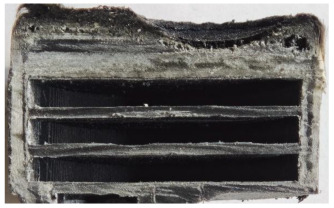	−0.091 ± 0.0002 mm/s	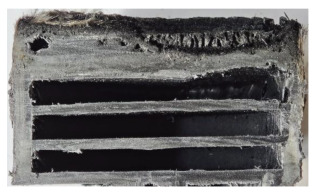	−0.122 ± 0.0002 mm/s
5.1		5.2	
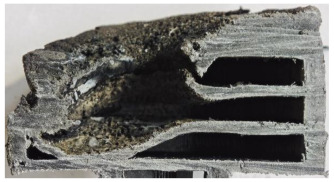	N/A	N/A	N/A
6.1		6.2	
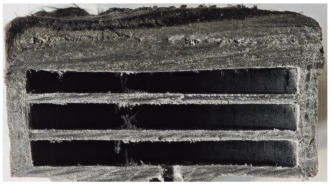	−0.036 ± 0.0002 mm/s	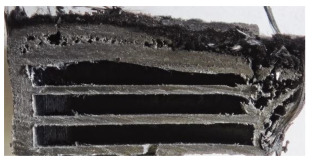	−0.040 ± 0.0002 mm/s
7.1		7.2	

## Data Availability

The original contributions presented in the study are included in the article, further inquiries can be directed to the corresponding author.
